# A comprehensive analysis of the hub genes for oxidative stress in ischemic stroke

**DOI:** 10.3389/fnins.2023.1166010

**Published:** 2023-05-09

**Authors:** Qing Zhou, Yang Dong, Kun Wang, Ziyan Wang, Bingquan Ma, Bo Yang

**Affiliations:** ^1^Rehabilitation Medicine, The First Affiliated Hospital of Zhengzhou University, Zhengzhou, China; ^2^Department of Neurosurgery, The First Affiliated Hospital of Zhengzhou University, Zhengzhou, China; ^3^Department of Anesthesiology, The First Affiliated Hospital of Zhengzhou University, Zhengzhou, China

**Keywords:** oxidative stress, ischemic stroke, antioxidant, therapy, prognosis, hub genes

## Abstract

Ischemic stroke (IS), resulting from the occlusion of the cerebral artery and subsequent interruption of blood flow, represents a major and critical threat to public health. Oxidative stress (OS) has been confirmed to play a role in the IS pathological process and neural death. Understanding the essential role of OS-related genes in ischemic stroke is critical to understanding the current perception of the pathophysiological process in IS. Herein, by integrating three IS datasets (GSE16561, GSE22255, and GSE58294), we divided IS samples into the low- and high-OS groups by calculating the OS score identified by the oxidative stress gene set. The functional enrichment analysis of differentially expressed genes (DEGs) between the low- and high-OS groups indicated that DEGs were associated with hypoxia, the inflammatory response, and oxidative phosphorylation pathways. Furthermore, nine hub genes (namely TLR1, CXCL1, MMP9, TLR4, IL1R2, EGR1, FOS, CXCL10, and DUSP1) were identified through the Girvan–Newman algorithm and cytoHubba algorithms. Nine hub genes were highly expressed in IS samples and positively related to neutrophils and macrophages. Drug-sensitive analysis targeting hub genes defined allopurinol and nickel sulfate as potential candidates for impairing the neural death caused by oxidative stress in IS. Finally, we employed five machine learning methods to check the efficacy of the predictive model identified by nine hub genes. The results showed that our model had superior power for predicting the OS activity of IS patients. TLR4 was found to have excellent diagnostic value and a wide-spectrum interaction with other hub genes. Our research emphasized the impact of oxidative stress on ischemic stroke, which supports the idea that antioxidants hold great promise in ischemic stroke therapy.

## 1. Introduction

Stroke is the main cause of death and long-term disability worldwide. Ischemic stroke (IS) is the primary type of stroke, accounting for more than 80% of all types of strokes (Cui et al., 2021; Jolugbo and Ariëns, 2021). Ephemeral or prolonged cerebral artery occlusion, followed by hypoxia, can lead to neuronal apoptosis and death, resulting in focal brain damage and functional defects, which are the major contributors to stroke-related morbidity and mortality (Campbell and Khatri, 2020; Tao et al., 2020). IS blocks the blood and oxygen supply to the brain, which induces a series of downstream metabolism events in the oxygen-rich tissues and neural cells (An et al., 2021), of which neuronal apoptosis, inflammatory response, angiogenic edema, and increased intracranial pressure occur. Recent studies have shown that chronic inflammation and blood–brain barrier leakage damage brain tissues (Spychala et al., 2018). At present, the effective treatment of ischemic stroke, including the intravenous injection of recombinant tissue plasminogen activator and intravascular thrombectomy, shows good therapeutic effects in recanalization (Paul and Candelario-Jalil, 2021). However, two rapid reperfusion methods are limited by extremely narrow treatment time windows, and only a minority of patients obtain timely treatment due to the strict contraindications (An et al., 2021). The pathophysiological mechanism underlying IS remains poorly defined. Therefore, there is an urgent need to delineate the mechanistic aspects and develop new treatment methods for improving the clinical outcome of IS.

A growing body of evidence has found that there is a continuum of intricate processes that play a role in neuronal death, such as neuroinflammation, oxidative stress, excitotoxicity, and apoptosis, in IS development (Li et al., 2017; Xiong et al., 2018; Qin et al., 2022). Oxidative stress (OS) is defined as a dysfunction between the generation of oxidants, reactive oxygen free radicals (ROS), and their abolishing system, antioxidants, under the harmful stimulation of internal and external environments (Ornatowski et al., 2020). Numerous reports have discovered that OS is associated with multiple diseases, as it produces excessive ROS that overwhelms the antioxidant system's maximum capacity (Zhang et al., 2020; Forman and Zhang, 2021). The overwhelming ROS levels hasten the oxidation of macromolecules, including nucleic acids, membrane lipids, and proteins, which is a damaging signal that leads to cellular dysfunction. Oxidative stress plays a crucial role in the pathogenesis of stroke by triggering a cascade of events, such as oxidative damage to lipids, proteins, and nucleic acids, leading to cytotoxicity (ref PMID 23011809**)**.

Additionally, it induces an inflammatory response and causes neuronal apoptosis, leading to neurodegeneration, and cell death. Moreover, it activates the autophagic pathway and damages the blood–brain barrier, further exacerbating the severity of the stroke (Ref PMID 29087944 and 36439687**)**. Given the devastating effects of oxidative stress in stroke, antioxidants have been proposed as a potential therapeutic approach to alleviate the pathological processes associated with this condition. The macrophage-mimicking MnO_2_ nanoparticles can scavenge the surplus ROS by recognizing adhesion molecules that interact with the macrophage membrane protein. The reduced oxidative stress signal modifies the inflammatory phenotypes by increasing the M2-macrophage amount, which facilitates the survival of injured neurons (Li et al., 2021). These findings have revealed that oxidative stress exerts an overarching influence on IS initiation, and antioxidant therapy could be a novel therapeutic approach for treating the reperfusion injury associated with IS.

The current study investigated the crosstalk between oxidative stress and IS progression and therapy. Three IS-associated GEO datasets (GSE16561, GSE22255, and GSE58294) and the oxidative stress gene set derived from the gene ontology website (http://geneontology.org/) were enrolled in our analysis work. We dissected the OS-related hub genes that play a crucial role in IS development. Similarly, the immune cell features of hub genes were also investigated, and we constructed a prognosis model for evaluating the OS level. Furthermore, the potential drugs that target OS hub genes were also identified. Multiple machine-learning methods were used to check the efficacy of the prognostic model created by the hub genes. Our research highlighted the major influence of oxidative stress on ischemic stroke, providing novel therapeutic opportunities for the treatment of IS.

## 2. Materials and methods

### 2.1. Data collection

Three IS GEO datasets, GSE16561 (24 control samples and 39 IS samples), GSE22255 (20 control samples and 20 IS samples), and GSE58294 (23 control samples and 69 IS samples), were selected for our analysis. The whole blood mRNA expression data in ischemic stroke patients and survival data were downloaded from the GEO website (https://www.ncbi.nlm.nih.gov/geo/). All the data processing and analysis were implemented in the R project. The original expression data were transformed into log2 format after background correction. Finally, three datasets were combined, and batch effects were eliminated by applying the “Combat” algorithm. In addition, the OS-related gene set was collected from the gene ontology database (http://geneontology.org/).

### 2.2. Identification of high- and low-OS groups

The single sample gene set enrichment analysis (ssGSEA) was employed to calculate the normalized enrichment score (NES), which represents the relative degree of OS level in each IS patient (Zhuang et al., 2021). Then, the total IS samples were divided into the high- and low-OS groups using hierarchical clustering based on the median OS score.

### 2.3. Function enrichment analysis

The DEGs between the high- and low-OS groups were acquired using the limma package in R with the significant criteria: |Log FC| of >0.5 and a *P*-value of < 0.05. Subsequently, the hallmark pathway set and the Kyoto Encyclopedia of Genes and Genomes (KEGG) pathway set were obtained from the online website (http://gsea-msigdb.org/) for the subsequent function enrichment analysis. The Clue GO tool in the Cystoscape software was introduced to visualize the pathway enrichment results. We selected the gene set enrichment analysis (GSEA) to explore the differential pathways between the high- and low-OS groups.

### 2.4. Immune infiltration cell analysis

CIBERSORT was developed to calculate the content of each immune cell subgroup by collecting the gene expression features of 22 human immune cell subtypes via the deconvolution method according to the principle of linear support vector regression (Newman et al., 2015). CIBERSORT is a superior method for the deconvolution analysis of complex mixtures and expression matrices containing similar cell types. The immune cell content of IS patients was evaluated by applying the CIBERSORT algorithm. Moreover, the differences in immune cells between the high- and low-OS groups were analyzed.

### 2.5. Establishment of hub genes

The protein-protein interaction (PPI) network of the aforementioned DEGs was obtained from the string website, and the key node genes were analyzed. Subsequently, the significant gene communities were identified using the Girvan–Newman algorithm (Newman and Girvan, 2004). A total of 12 genes in 18 significant gene communities were found. Furthermore, we acquired the top 10 genes calculated by three cytoHubba algorithms (MCC, MNC, and degree) (Han et al., 2021). The overlapping genes between the 12 community genes and the top 10 genes were identified as the hub genes. Nine hub genes were acquired for the following analysis.

### 2.6. Correlation analysis of hub genes and immune cells

Based on our metadata generated by three GEO datasets, we investigated the expression differences of nine hub genes in the normal group and IS samples. Subsequently, the correlation between the gene expression of 9 hub genes and 22 immune cells was analyzed using CIBERSORT.

### 2.7. Sensitive drug analysis of hub genes

The sensitive drug candidates of nine hub genes were investigated using the DSigDB database in Enrichr (https://maayanlab.cloud/Enrichr/).

### 2.8. Prognostic value analysis of hub genes

Five machine learning methods, including logistic, Bayesian logistic, decision tree, random forest, and boosting, were used to evaluate the predictive accuracy of the hub gene model in diagnosing the IS samples with different OS statuses. The predominant hub genes were screened out by boosting. Furthermore, the receiver operating curves were used to examine the predictive value of nine hub genes in estimating the OS level. Finally, the correlation analysis between the nine hub genes and each other was conducted to determine the key node hub genes.

## 3. Results

### 3.1. Identification of the high- and low-OS groups in IS

**Figure 1:**
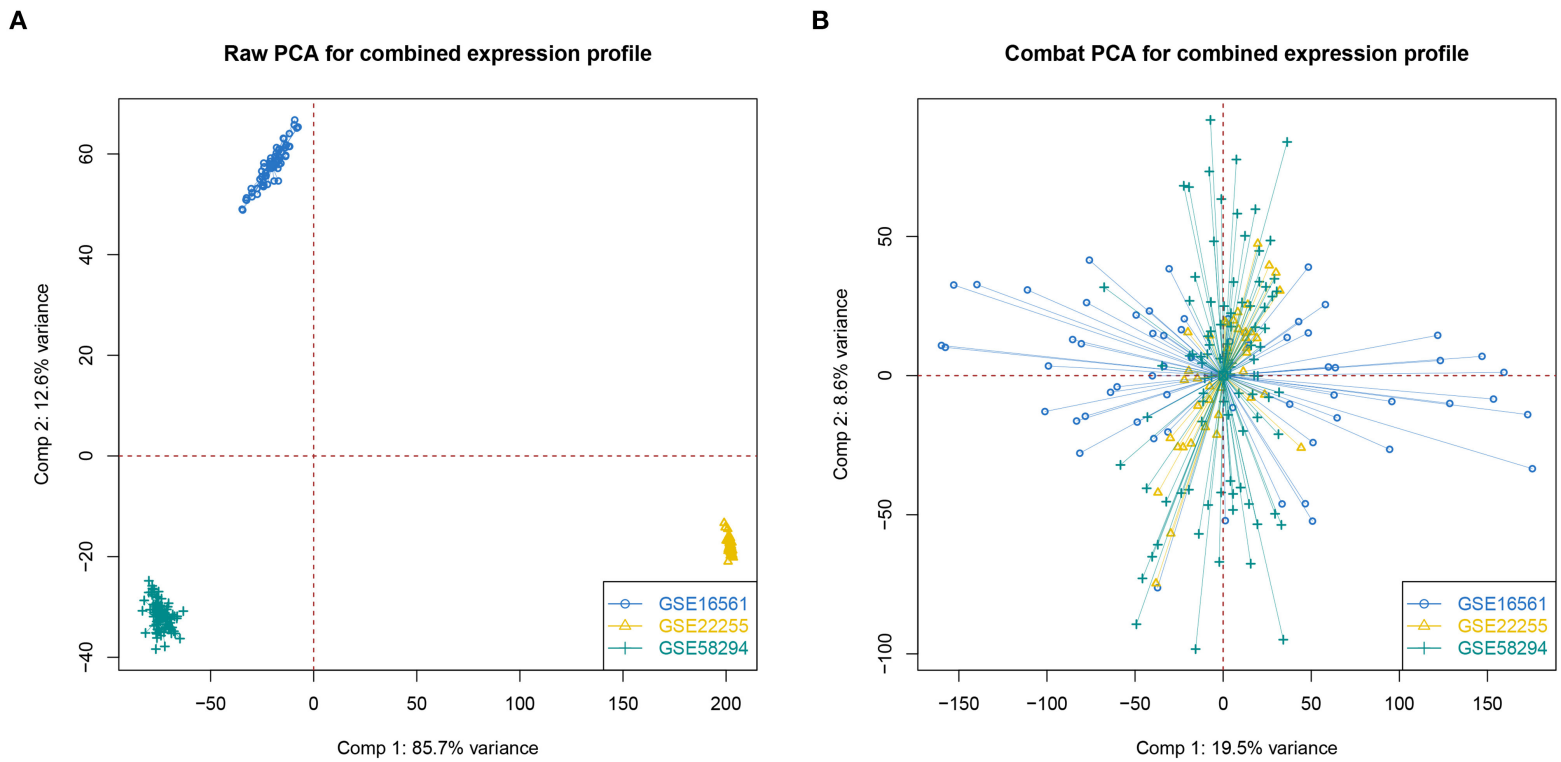
The PCA analysis of data merging. **(A, B)** PCA analysis without or with correcting batch effect.

**Figure 2:**
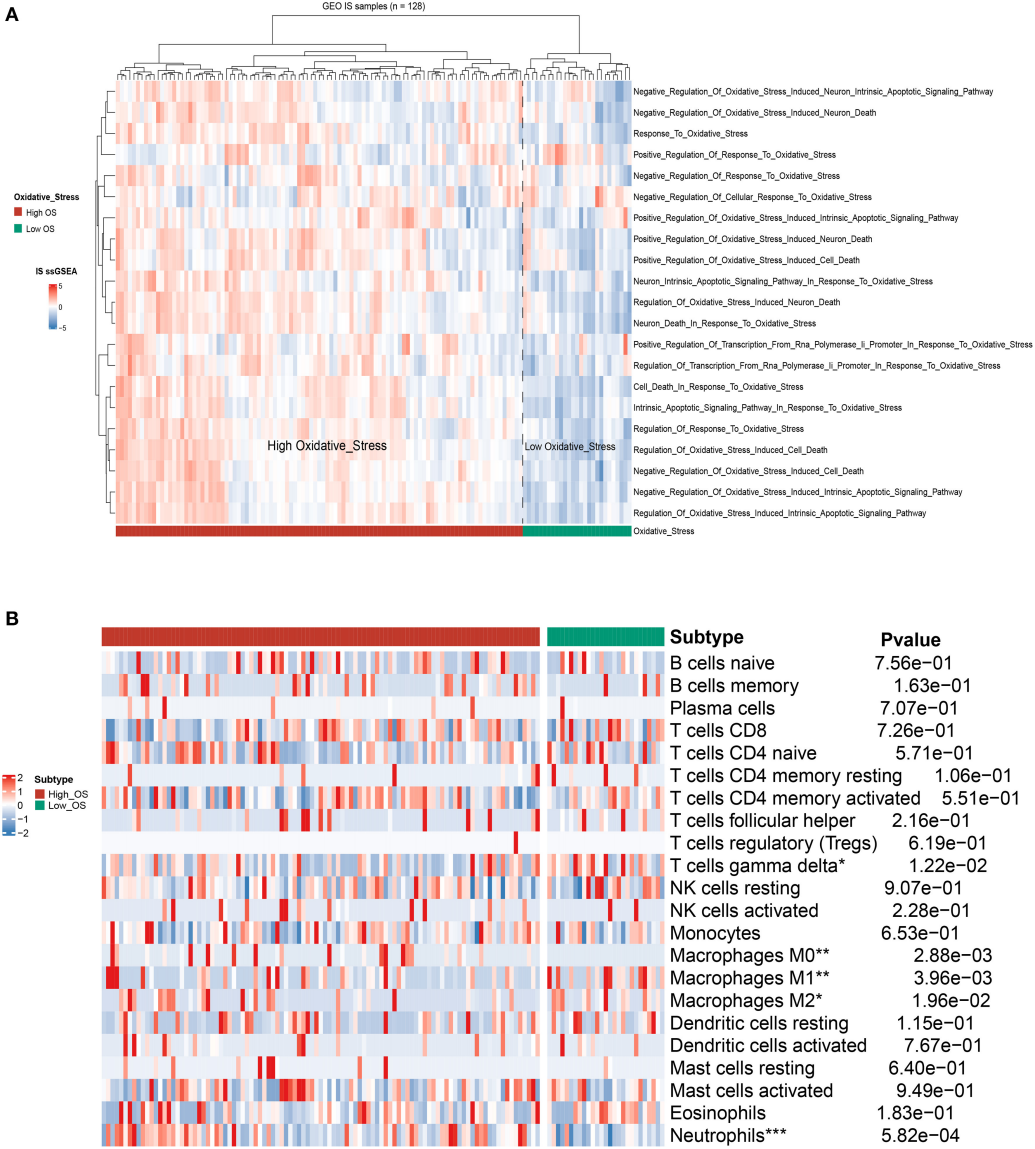
The identification of OS subtypes based on ssGSEA. **(A)** The hierarchical clustering heat map of the high- and low-OS groups. **(B)** The heatmap of immune cell flirtation in two OS subgroups. *p < 0.05, **p < 0.01, and ***p < 0.001 indicated the statistical significance of data.

First, we merged three IS-associated GEO datasets (GSE16561, GSE22255, and GSE58294) into the metadata for the following comparable analysis. The sample distribution pattern is shown in Figure 1A before data merging, while Figure 1B displays the PCA result after discharging the batch effect, suggesting that the samples were mixed thoroughly. Single sample Gene Set Enrichment Analysis (ssGSEA) indicated that 128 IS samples were divided into the high- and low-OS groups (Figure 2A). The contents of neutrophils and macrophages in the high-OS group were significantly increased, indicating that oxidative stress influenced the neutrophils and macrophages in IS (Figure 2B).

### 3.2. GSEA and KEGG analysis between low- and high-OS groups

**Figure 3:**
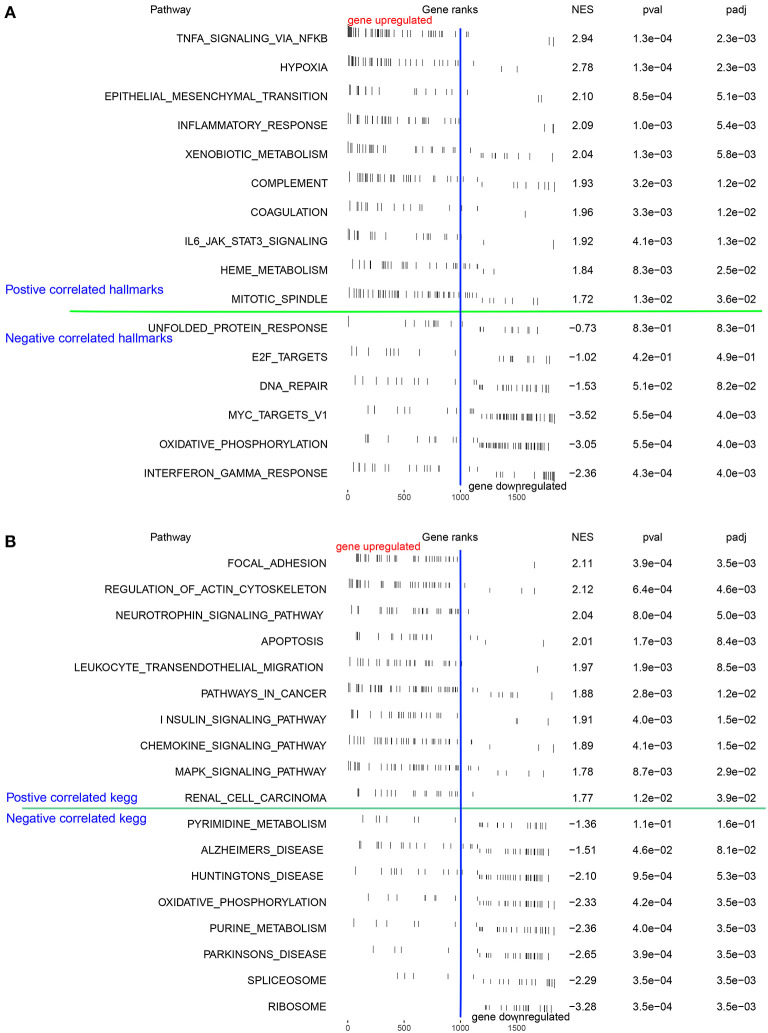
Function enrichment analysis. **(A, B)** The GSEA and the KEGG analysis of DEGs between the high- and low-OS groups.

We examined the differentially expressed pathways between the high- and low-OS groups using GSEA. The investigation from the hallmark gene sets showed that hypoxia, TNF signaling through the NF-κB pathways, epithelial-mesenchymal transition, and inflammatory response were significantly concentrated in the high-OS group, while the oxidative phosphorylation and interferon-γ response pathways were augmented considerably in the low-OS group (Figure 3A). The KEGG pathway analysis demonstrated that focal adhesion, regulation of the actin cytoskeleton, the neurotrophic signaling pathway, apoptosis, and leukocyte migration through endothelial cells, and the chemokine signal transduction pathway were substantially enriched in the high-OS group, suggesting that the high oxidative stress activity was associated with inflammatory response (Figure 3B). The low-OS group was characterized by the RNA processing pathways such as spliceosomes and ribosomes (Figure 3B). As the level of oxidative stress increased, the levels of hypoxia and inflammation in patients with IS also increased.

### 3.3. Identification of hub genes

**Figure 4:**
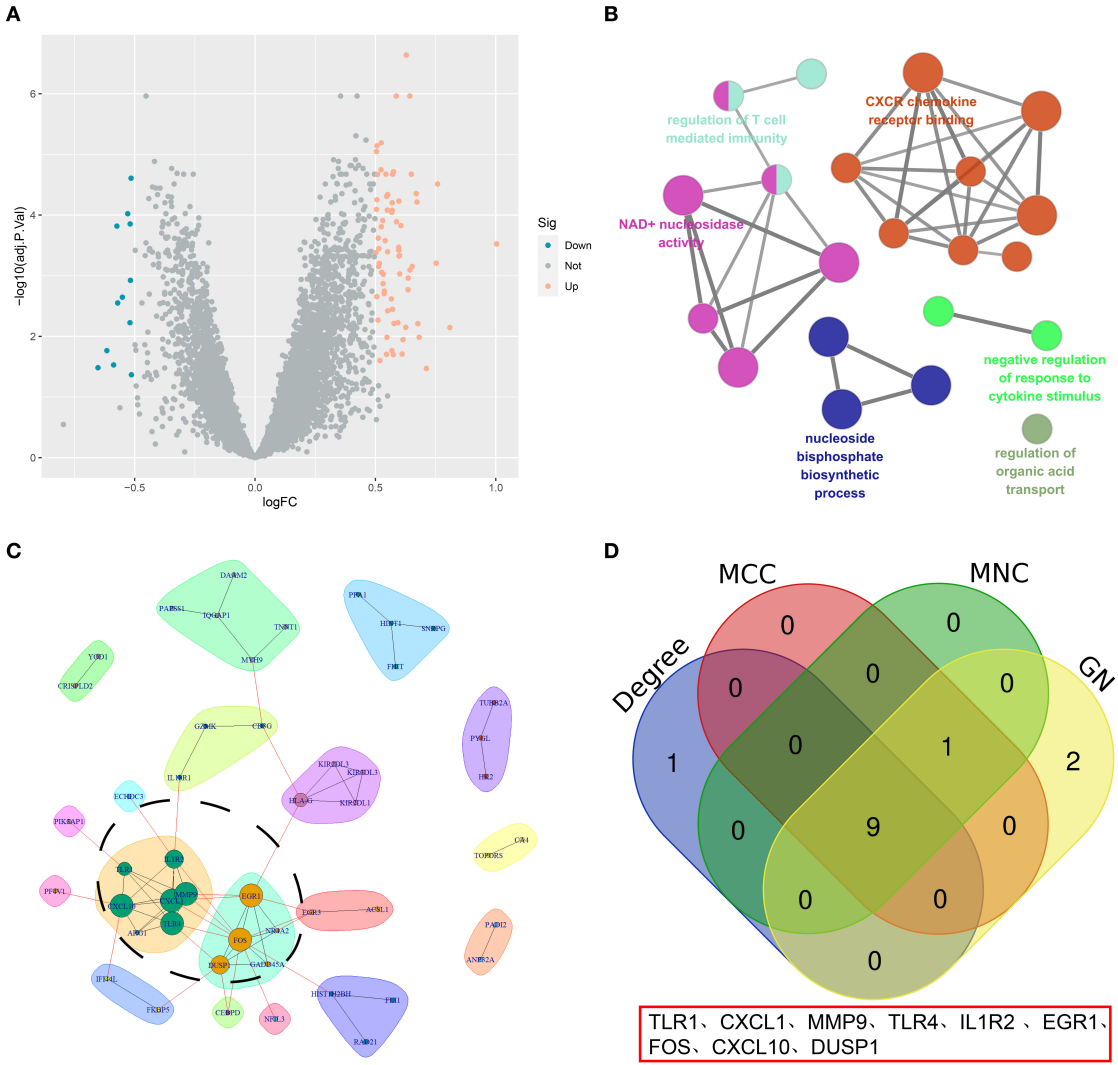
The screening of hub genes. **(A)** A volcano map of DEGs between the high- and low-OS groups. **(B)** The KEGG analysis of DEGs. **(C)** The significant gene community identified by GN. **(D)** The Venn diagram of hub genes.

A total of 85 DEGs between the high- and low-OS groups were collected (Figure 4A). The Clue GO enrichment analysis indicated that these DEGs were significantly associated with NAD+ nucleosidase activity and the CXCR chemokine receptor binding signaling pathways (Figure 4B). The genes of significant communities were obtained using the Girvan–Newman algorithm and 18 gene communities, among which 12 vital genes were found (Figure 4C). We also acquired the top 10 genes identified by the three cytoHubba algorithms (MCC, MNC, and Degree). Taking the section between 12 community genes and 10 top genes, nine overlapping genes (namely TLR1, CXCL1, MMP9, TLR4, IL1R2, EGR1, FOS, CXCL10, and DUSP1) were regarded as hub genes for the following analysis (Figure 4D).

### 3.4. Correlation of hub genes and immune infiltration cells

**Figure 5:**
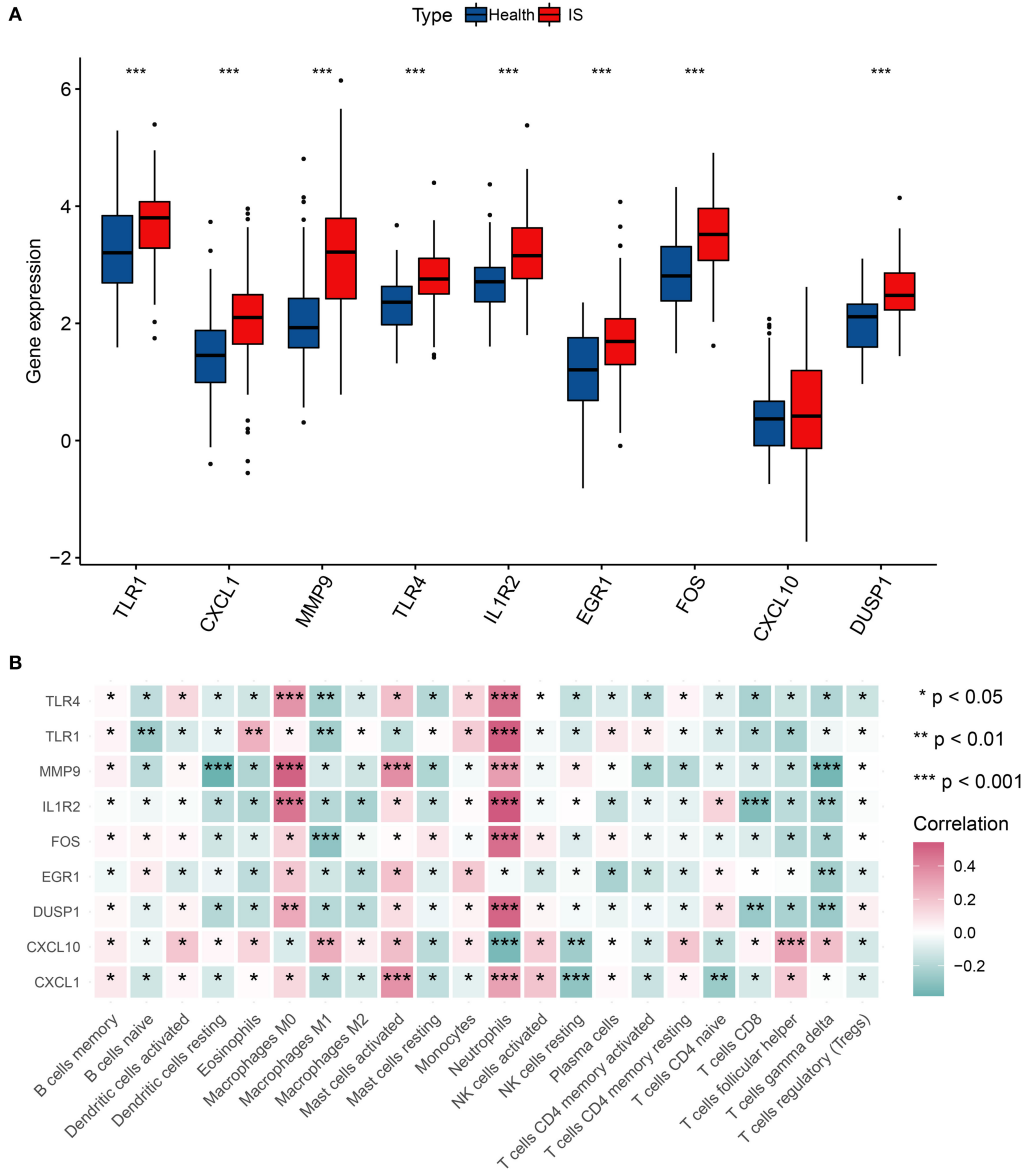
Correlation analysis of hub genes and immune cells. **(A)** The gene expression of nine hub genes in IS. **(B)** Correlation matrix of nine hub genes and immune infiltration cell amount. *p < 0.05, **p < 0.01, and ***p < 0.001 indicated the statistical significance of data.

First, we surveyed the overall gene expression pattern of nine hub genes in IS samples and matched the normal groups. The results showed that all hub genes other than CXCL10 were enhanced in IS patients, suggesting that these hub genes played a prompting role in IS initiation (Figure 5A). Most hub genes were positively correlated with neutrophils, M0-macrophages, and activated mast cells, representing a severe inflammatory response. However, there was an overall inverse correlation between most hub genes and T cells with distinct functional phenotypes (Figure 5B).

### 3.5. The potential sensitive drug prediction of hub genes

**Figure 6:**
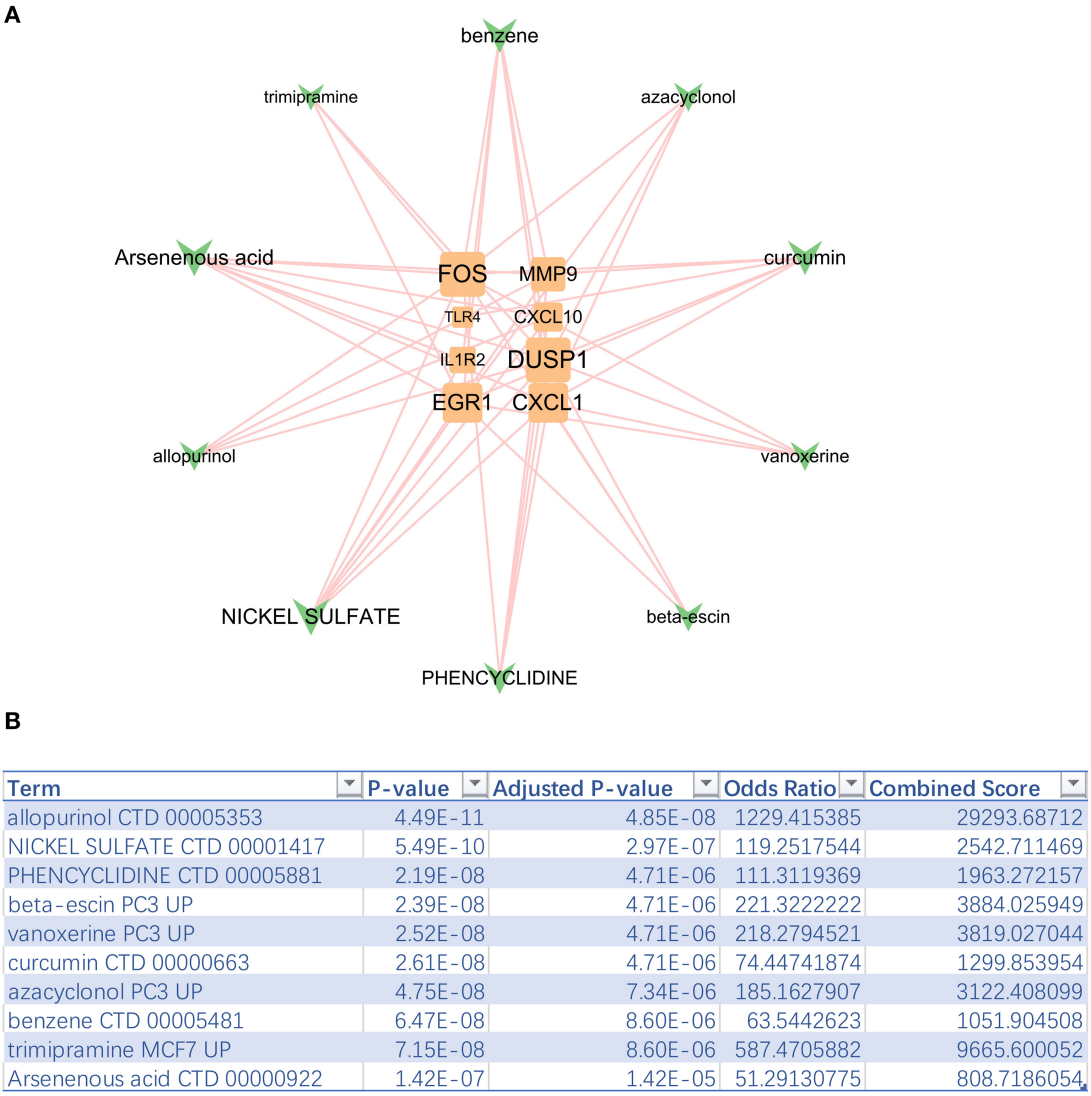
Top 10 sensitive drugs targeting hub genes. **(A)** The hub gene-drug network. **(B)** Top 10 sensitive drugs with the highest combined scores.

The top 10 sensitive drugs were found to be allopurinol, nickel sulfate, phencyclidine, beta-escin, vanoxerine curcumin, azacyclonol, benzene, trimipramine, and arsenenous acid (Figures 6A, B). It was found that allopurinol and nickel sulfate were sensitive to nearly all hub genes. Developing a novel strategy based on allopurinol and nickel sulfate could improve the clinical outcome of IS patients with the genetic hub gene features. Notably, FOS, EGR1, DUSP1, and CXCL1 were discovered to have a strong interactive relationship with the sensitive drugs compared with other hub genes.

### 3.6. Prognostic effect validation of hub genes

**Figure 7:**
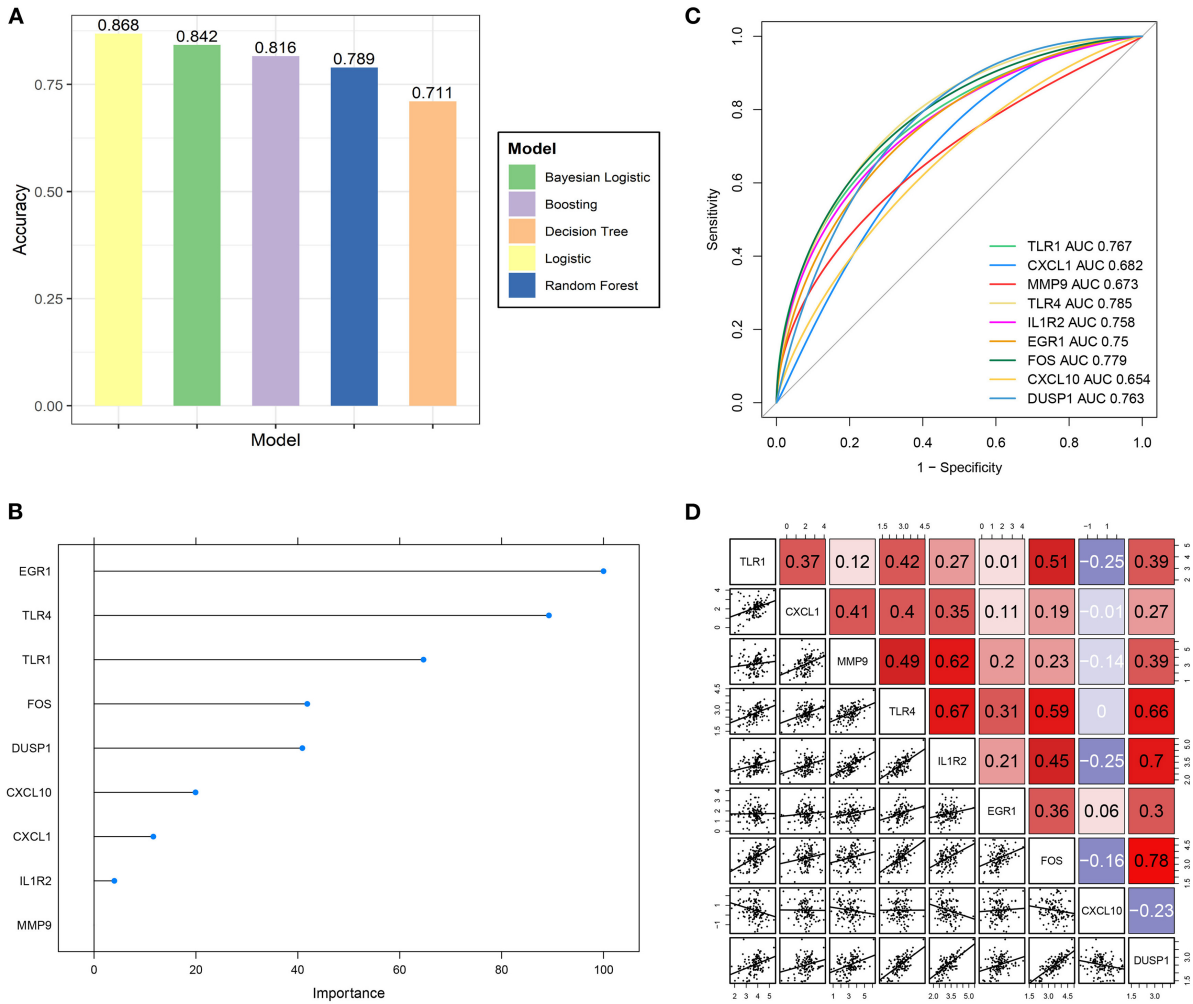
Predictive ability validation of hub genes. **(A)** The predictive accuracy of the prognostic model by five machine learning methods for the IS OS subgroups. **(B)** The importance ranking of nine hub genes by boosting analysis. **(C)** The ROC curve of nine hub genes predicts the high- and low-OS groups. **(D)** Correlation analysis of nine hub genes with each other.

Multiple machine-learning methods were employed to evaluate the predictive accuracy of hub genes in distinguishing the IS subtypes with different OS levels. The results from five machine learning methods showed that the model composed of nine hub genes could distinguish the high-OS group from the low-OS group (Figure 7A). The prognostic model generated by nine hub genes performed excellently in evaluating the OS status. Boosting results showed that EGR1, TLR4, and TLR1 were three important factors in the prognostic signature (Figure 7B). Among the nine hub genes, TLR4 had the highest AUC value (Figure 7C). At the same time, there was a good positive correlation between TLR4 and the other eight hub genes (Figure 7D).

## 4. Discussion

The current study divided the IS samples into the low- and high-OS groups according to the median oxidative stress score calculated using the ssGSEA method. In this study, we found that hypoxia signaling was mainly enriched in the high oxidative stress group compared with the low oxidative stress activity group, suggesting the intricate interaction between hypoxia and the OS response. A previous study reported that hypoxia is a predominant driving factor in inducing oxidative stress (Pialoux and Mounier, 2012). For example, hypoxia exposure mediates oxidative stress in the brain region by reducing the biochemical activity of antioxidant enzymes such as SOD, CAT, and GSH while elevating oxidative stress markers such as MDA and TAC in the hippocampus (Mohamed et al., 2019). The periodic aggregation of HIF-1α induced by hypoxia promotes NADPH oxidase activation, intensifying ROS export (Prabhakar and Semenza, 2012; Wang et al., 2020).

Conversely, oxidative stress governs the hypoxia process. The latest proceedings have certified that high-ROS concentration stimulation increases HIF's expression (You et al., 2021). As previously described, OS swells the retinal cell inflammation by activating the cGAS-STING signaling provoked by inflating DNA damage and cytosolic leakage (Zou et al., 2022). We also found that the inflammation signal pathway was augmented in the high-OS group of IS patients, highlighting the prevailing function of oxidative stress in controlling inflammation. Consistent with our findings, the KEGG analysis revealed that leukocyte transendothelial migration, the chemokine signaling pathway, and chemokine receptor binding were augmented in the high-OS group, demonstrating the active inflammatory response in the context of high oxidative stress.

By performing the Girvan–Newman analysis, we identified 12 key genes in 18 predominant gene communities. We further identified nine overlapping hub genes, including TLR1, CXCL1, MMP9, TLR4, IL1R2, EGR1, FOS, CXCL10, and DUSP1, by taking the intersection between the 12 community genes and the top 10 genes defined by three cytoHubba algorithms. Our analysis revealed that these hub genes play crucial roles in the oxidative stress process, which is often associated with inflammation and thrombosis.

For instance, a previous study has shown that an excess concentration of hepatic CXCL1 leads to an oxidative stress response, as indicated by the increased activation of stress kinases such as apoptosis signal-regulating kinase 1, which accelerates the nonalcoholic steatohepatitis progression (Hwang et al., 2020). TLR4 activation can increase the oxidative stress response and enhance ROS generation in the activated macrophage cells in thrombosis, while nattokinase treatment can reduce inflammation and oxidative stress (Wu et al., 2020).

Additionally, our correlation analysis indicated that hub gene expression levels were positively associated with the numbers of neutrophils and macrophages, which are the major infiltration cells in the inflammatory response (Martini et al., 2019). There was a complex crosstalk between oxidative stress and inflammation in ischemic stroke. We also found evidence that the deficiency of S-adenosylhomocysteine hydrolase (SAHH) urges the EGR1 to recruit in the promoter region of the thioredoxin-interacting protein (TXNIP), which increases TXNIP expression (Dai et al., 2021). The excessive TXNIP signal promotes oxidative stress and follows NLRP3 inflammasome motivation, contributing to diabetic nephropathy progression (Dai et al., 2021). Our analysis demonstrated that these hub genes are involved in the oxidative stress process, with nearly all hub genes being upregulated in IS samples relative to the normal groups. Taken together, our findings suggest that developing anti-thrombotic drugs with anti-inflammatory and antioxidative stress effects could be a promising therapeutic strategy for ischemic stroke.

The management of acute ischemic stroke at present involves the crucial process of mitigating the harmful effects of excessive ROS during ischemia/reperfusion. One protective microglia subtype in stroke-associated microglia is characterized by the upregulation of an antioxidant enzyme, Peroxiredoxin-1 (Prdx1), which induces the expression of stroke-protective molecules, such as osteopontin and ferritin (Kim et al., 2022). Inhibiting Prdx1 expression significantly intensifies the infarction and inflammatory responses by suppressing the antioxidant gene, such as Txn1 and Mt2 expression (Kim et al., 2022). With antioxidants being a part of current IS treatment, we sought to identify potential drugs targeting hub genes. Among the 10 drugs found to be sensitive, allopurinol and nickel sulfate were discovered to be sensitive to almost all hub genes. Finally, we constructed a predictive model depending on nine hub genes to evaluate oxidative stress activity in ischemic stroke. The analysis from five machine learning methods (logistic, Bayesian logistic, decision tree, random forest, and boosting) showed that the predictive model consisting of nine hub genes exhibited excellent performance. EGR1, TLR4, and TLR1 were identified as the three key factors in determining the oxidative stress subgroups, which is consistent with the results of previous studies (Wu et al., 2020; Dai et al., 2021). We discovered that TLR4 had the highest AUC value and the strongest positive correlation with the other eight hub genes. Increasing TLR4/NOX2 signaling activity triggers a severe oxidative stress response in polystyrene microplastics-mediated uterine fibrosis (Wu et al., 2022). Suppressing the TLR4/NOX2 signaling pathway significantly decreases ROS export in cells and curbs the expression of fibrotic and collagen-associated genes (Wu et al., 2022). Antioxidant therapy targeting TLR4/NOX2 signaling could be an innovative option for alleviating uterine fibrosis. Together with our data, these findings highlight the critical role of TLR4 in oxidative stress and antioxidant therapy.

## 5. Conclusion

In summary, our study provides valuable insights into the role of oxidative stress in the pathological process of ischemic stroke. Two IS subgroups were formed based on the high- and low-OS levels. Significant differences were observed in the expression levels of genes related to hypoxia and inflammation between the two groups. Notably, nine hub genes which are primarily associated with neutrophils and macrophages were found to be significantly upregulated in the IS samples. Using machine learning algorithms, we developed a predictive model based on nine hub genes. It can potentially facilitate the development of novel therapeutic targets for improving the clinical outcome of ischemic stroke. Our findings contribute to a better understanding of the underlying mechanisms of ischemic stroke and may lead to the development of more effective interventions for this clinically important condition.

## Data availability statement

The datasets presented in this study can be found in online repositories. The names of the repository/repositories and accession number(s) can be found in the article/supplementary material.

## Author contributions

QZ, YD, and KW contributed to the design and implementation of the research and wrote the manuscript. ZW contributed to the analyses of the results. QZ wrote the article and provided critical comments. BM and BY designed and supported the study and edited the manuscript. All the authors substantially contributed to the work presented in this article. All authors reviewed and approved the final manuscript.
